# Focal Distribution of Hepatitis C Virus RNA in Infected Livers

**DOI:** 10.1371/journal.pone.0006661

**Published:** 2009-08-18

**Authors:** J. David Stiffler, Minhhuyen Nguyen, Ji A. Sohn, Chen Liu, David Kaplan, Christoph Seeger

**Affiliations:** 1 Fox Chase Cancer Center, Philadelphia, Pennsylvania, United States of America; 2 University of Florida, Gainesville, Florida, United States of America; 3 Research Section, Philadelphia Veterans Administration Medical Center, Philadelphia, Pennsylvania, United States of America; 4 Gastroenterology Division, University of Pennsylvania, Philadelphia, Pennsylvania, United States of America; Yale University, United States of America

## Abstract

**Background:**

Hepatitis C virus (HCV) is a plus-strand RNA virus that replicates by amplification of genomic RNA from minus strands leading to accumulation of almost one thousand copies per cell under *in vitro* cell culture conditions. In contrast, HCV RNA copy numbers in livers of infected patients appear to be much lower, estimated at a few copies per cell.

**Methodology/Principal Findings:**

To gain insights into mechanisms that control HCV replication *in vivo*, we analyzed HCV RNA levels as well as expression of interferon beta (IFNβ) and several interferon stimulated genes (ISGs) from whole liver sections and micro-dissected subpopulations of hepatocytes in biopsy samples from 21 HCV-infected patients. The results showed that intrahepatic HCV RNA levels range form less than one copy per hepatocyte to a maximum of about eight. A correlation existed between viral RNA levels and IFNβ expression, but not between viral RNA and ISG levels. Also, IFNβ expression did not correlate with ISGs levels. Replication of HCV RNA occurred in focal areas in the liver in the presence of a general induction of ISGs.

**Conclusion/Significance:**

The low average levels of HCV RNA in biopsy samples can be explained by focal distribution of infected hepatocytes. HCV replication directly induces IFNβ, which then activates ISGs. The apparent lack of a correlation between levels of IFNβ and ISG expression indicates that control of the innate immune response during HCV infections depends on multiple factors.

## Introduction

Hepatitis C virus (HCV) is a major human pathogen that chronically infects an estimated 180 million people worldwide [1]. HCV replicates in hepatocytes, which then become the target of an inflammatory response that leads to progressive fibrosis of the liver, culminating in cirrhosis and often hepatocellular carcinoma, both of which cause significant morbidity and mortality. In the United States, HCV infection is the predominant cause for liver failure and orthotopic liver transplantations (OLT). However, near-universal graft re-infection results in accelerated fibrosis and cirrhosis of the allograft [2]. While a cure from HCV infections can be achieved in approximately 50% of infected patients with interferon-alpha (IFNα)-based therapy, the mechanism by which therapy can cure patients remains unclear.

HCV is a plus-strand RNA virus belonging to the Flaviviridae family of viruses, which include several other human pathogens such as West Nile (WNV) and Dengue viruses [3]. As with other plus-strand RNA viruses, HCV infections in tissue culture cells result in rapid amplification of the viral genome leading to the accumulation of several hundred to a few thousand genomes per cell [4], [5]. HCV replication in tissue culture cells is very sensitive to treatment with IFNα/β and IFNγ [6], [7], [8]. Similarly, HCV replication is also inhibited *in vivo* by IFNα, although sensitivity to the cytokine varies among patients for reasons that are not well understood [9], [10].

The discovery that HCV infections in chimpanzees and humans induce expression of genes that are normally activated by IFNs, could explain why HCV RNA and protein levels are very low in infected livers [11], [12], [13], [14]. Consistent with this view, a study with experimentally infected chimpanzees revealed that on average liver cells carry less than 10 copies of HCV plus strand RNA [15]. Similarly, PCR-based RNA analyses of RNA isolated from biopsy samples from infected human patients suggested the presence of only very low average copy numbers [16]. By contrast, several studies using less sensitive *in situ* hybridization methods for the detection of viral plus- and minus-strand RNA nonetheless provided evidence for much more robust HCV RNA replication levels [17], [18]. In addition to these conflicting results regarding the overall levels of HCV RNA replication in infected livers, the distribution of infected hepatocytes, e.g. whether HCV replicates in a subpopulation of cells as efficiently as observed in tissue culture cells, or whether it replicates in a large fraction of hepatocytes with a few copies of RNA per cell, remains unknown. The first scenario might suggest a model in which ISGs are globally activated but locally repressed (i.e. by the virus) to allow efficient viral replication in isolated pockets; the second possibility would invoke a model in which amplified RNA is rapidly degraded due to the highly up-regulated ISGs. The second model would predict a correlation between viral RNA load in the liver and activation of ISGs. Clarification of whether focal inactivation of ISGs can explain persistence of HCV infections could have an impact on the design of future antiviral therapies.

To determine the distribution of HCV and ISG expressing cells, we have performed gene expression analyses on frozen liver sections from HCV infected patients. We found that only a subpopulation of hepatocytes expresses detectable levels of HCV RNA, while cells expressing ISGs are more uniformly distributed in the liver. Consistent with previous PCR-based studies, we estimated that the average copy number per hepatocyte ranges from less than one to about 10 copies of viral RNA per hepatocyte.

## Materials and Methods

### Liver tissue

Liver tissues were obtained from patients undergoing liver needle biopsies for diagnostic purposes. All patients signed consent forms to acknowledge participation in this study approved by the institutional review board of the Fox Chase Cancer Center, Philadelphia, the University of Pennsylvania, Philadelphia and the University of Florida, Gainesville. Fractions of the core of the needle biopsies were snap frozen in liquid nitrogen immediately following the procedures, embedded in OTC and stored at −80°C. Frozen sections were prepared with a cryostat (CM1850, LEICA) set at 7 µm. Sections were mounted onto non-charged Premium Microscope slides (12-544-2, Fisher), placed immediately into a slide box on dry cry ice and stored at −80°C.

### Laser capture microdissection (LCM) and RNA isolation

Tissue sections were stained with hematoxylin/eosin using the HistoGene kit (Arcturus). Cells were captured with a laser capture microdissection microscope (Veritas, Arcturus) using HS caps (Arcturus). The power and pulse settings were adjusted to achieve a spot size of 22–24 µm. The number of hits was set to position 5. Isolation of total RNA from the captured cells was performed with the PicoPure RNA Isolation Kit (0204, Arcturus). For the isolation of total RNA from whole tissue sections and from HCV expressing GS4.1 cells the same protocol was followed.

### cDNA Synthesis and DNA amplification

The High Capacity cDNA Archive kit (4322171, ABI) was used for cDNA synthesis. cDNAs were pre-amplified with a PreAmp kit from ABI (4366381). The pooled TaqMan probe/primer mix consisted of a mixture of the TaqMan primers specific for the selected genes (Table S1). The reactions were performed in a thermocycler for 9 (whole sections) or 14 (LCM samples and GS4.1 RNA) cycles. The samples were diluted 1∶5 with 200 ul of TE and stored at –80°C. Note that the reactions did not contain the primer set for 18S RNA. The real-time PCR reactions were carried out in a total volume of 25 µl in an ABI7900 SDS instrument.

### Calculation of HCV copy numbers

**Figure 1:**
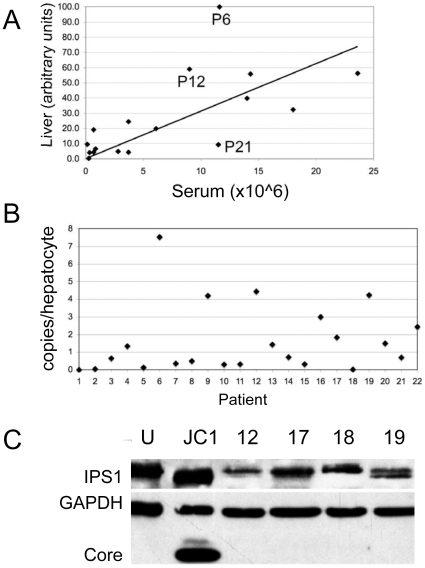
Correlation between HCV RNA in serum and liver. (A) Values for viral RNA in serum (x-axis) were plotted against HCV RNA levels (y-axis) in frozen sections from needle biopsies determined by quantitative real-time PCR (qRT-PCR). The values were normalized with albumin and expressed on a scale from 0 to 100. (B) The plot shows the estimated copy number of HCV RNA per hepatocyte for each of the 22 patients listed in Table 1. C) The figure shows a western blot analysis with protein extracts from normal, uninfected Huh7 cells (U), HCV JC1 infected Huh7 cells (JC1) and from liver tissues of patients 12, 17,18 and 19 (see Table 1). The blot was incubated with antibodies specific for the indicated proteins. Core, HCV core protein.

HCV copy numbers shown in Figure 1B were computed as follows: First, the Ct values obtained for HCV RNA levels were normalized with albumin with the following formula: 2^∧^-(Cthcv-Ctalb). Then, the calculated values from tissue culture cells (GS4.1) were divided by the values from liver tissues and multiplied by 1000, the estimated copy number of HCV RNA in GS4.1 cells, which yielded the number of HCV RNA copies/liver cell. This number was multiplied by 1.43 (1/0.7) to adjust for the 70% fraction of hepatocytes in liver tissue.

### Western blot

To prepare protein extracts, OTC was dissolved in PBS and the tissue sample transferred to lysis buffer (10 mM Tris (pH 8), 150 mM sodium chloride, 0.5% deoxycholic acid, 0.1% sodium dodecyl sulfate, 1% triton-X100) and protease inhibitor cocktail (Roche). Proteins (20 µg) were separated by 10% SDS-PAGE and transferred to Immobilon-P (Millipore). Membranes were probed with the indicated antibodies.

### Statistical analysis

Regression analysis was used to determine the significance of trend lines. To obtain cut points for positive samples in P2 and P4, the following formula was used: limit over which HCV is>null mean+1.96 std. The mean and standard deviations of the data for control (uninfected) patient P1 (null) were 0.793 and 1.088, respectively. The calculated value for the limit was 2.925. The list of entries for which HCV>2.925 included P2-13, P4-12, P4-14P, P4-23, P4-32, P4-34P, P4-41, P4-62 and P4-71P.

## Results

### Patient cohort and viral RNA titers

The cohort for this study consisted of patients chronically infected with HCV belonging to genotypes 1 (11/21), 2 (1/21), 3 (2/21) and 6 (5/21) (Table 1). One tissue sample (P1) was obtained from a colon cancer patient that was not infected with HCV, HBV or HIV and seven were derived from transplanted livers. Biopsies from patients 2–7 were obtained following OLT and during treatment with immunosuppressants. Patients 9 and 13 received IFN therapy prior to the liver biopsies. None of the patients received IFN therapy at the time of liver biopsy. Viral load (VL) in serum differed significantly among patients, ranging from about 1.2×10^∧^5 to 2.4×10^∧^7 genomes/ml. To investigate whether a correlation existed between viral load in blood and viral RNA in liver, we determined HCV RNA levels in tissue sections prepared from liver biopsies. To account for differences in the number of cells on histological sections used for RNA extraction, we normalized the values for HCV RNA levels with those for albumin. The results showed that the correlation between serum RNA and liver RNA levels was statistically significant (P = 3.63E-03) (Figure 1A). These results were consistent with those from a study by Vona et al. with a cohort of about 20 HCV infected patients [16]. Moreover, consistent with that study, we did not observe a correlation between HCV RNA levels and fibrosis or inflammation.

**Table 1:** Patient cohort.

*Patient*	*Race*	*GT*	*VL (x10^6^)*	*ALT*	*FIBR*	*INFL*	*RESPONSE*
P1		NA	0	ND	0	0	NA
P2*	C	3	ND	ND	0	2	NA
P3*	C	1	ND	ND	0	1	NA
P4*	C	1	ND	50	0	2	SVR
P5*	C	1	ND	ND	0	0-1	NA
P6*	C	1a	11.6	226	1	1	NA
P7*	B	1	2.8	573	0-1	1	NA
P8	B	1a	0.85	84	0-1	1	NA
P9	C	1a	14.3	3×UNL	3-4	2-3	RE
P10	B	1a	0.32	2×UNL	0	2-3	SVR
P11	A	1b	3.7	2×UNL	0	2	SVR
P12	A	1b	9.0	1-2×UNL	0	1-2	SVR
P13	C	3a	0.67	3-4×UNL	3-4	3-4	RR/SVR
P14	A	6a	0.12	2-3×UNL	4	4	RR/SVR
P15	C	2	0.67	2-3×UNL	3	2	RR/SVR
P16	A	6,HB	14.0	Normal	0	0-1	SVR
P17	A	1b	3.7	1.5×UNL	1	2	SVR
P18	C	1b	0.256	1.5×UNL	2	2	NA
P19	A	6	23.6	Normal	1	1-2	RR/SVR
P20	A	6	6.1	2×UNL	1	1-2	SVR
P21	C	1	11.5	2×UNL	2	2	ETR/RE
P22	A	6	18.0	Normal	1	1-2	RR/ETR/RE

* Patient underwent OLT prior to liver biopsy. HB, HBV co-infection; unl, upper normal limit; RE, relapse; RR, rapid response (HCV RNA (-) at 4–5 weeks of therapy; SVR, sustained viral response (HCV RNA (-) for >24 weeks post therapy); ETR, HCV RNA (-) at end of therapy; UNL, upper normal level, ND, not done; NA, not applicable; GT, HCV genotype, VL, viral load; ALT, (serum) alanine aminotransferase; FIBR, fibrosis (scoring range: 0–4 [31]), INFL, portal inflammation (scoring range 0–4 [31]).

To obtain an estimate for the copy number of HCV RNA in infected liver tissue, we compared HCV RNA levels in tissue culture cells expressing HCV subgenomic replicon (the Huh7-derived GS4.1 cell line [5]) with those in infected livers using values for HCV RNA and albumin obtained with the same RNA purification and qRT-PCR methods. We based our calculations on the assumption that albumin levels in the GS4.1 cells are comparable with those in hepatocytes *in vivo* and on information about the copy number of HCV RNA in those cells, which was estimated to be about 1000 per cell based on northern blot analysis [5]. Based on our calculations (see legend to Figure 1B), the copy number of HCV RNA in the livers of our patient cohort ranged from less than one copy per hepatocyte to a maximum of 7.5 copies in patient 6 (Figure 1B). Since the ratio between plus- and minus-strand RNA in HCV and other plus-strand RNA viruses is generally believed to be in the range of 10∶1 [4], our results indicated either that only a fraction of hepatocytes was infected or that the ratio of plus- to minus-strand RNA was much lower *in vivo* than observed in tissue culture cells or that a combination of the two possibilities existed.

The HCV protease NS3 cleaves the cellular protein IPS1, which leads to an inhibition of the signal transduction pathway for expression of IFNβ in response to viral infections [19], [20]. Therefore, cleaved IPS1 can be used as a reporter for HCV replication in infected cells. We have analyzed IPS1 by western blot analysis of protein extracts from normal and HCV infected tissue culture cells and from eight patient samples. The results showed that in infected tissue culture cells IPS1 is completely cleaved (Figure 1C). In contrast, in most patient samples IPS1 migrated as a full-length protein (Figure 1C, results not shown). The highest fraction of cleaved IPS1 was observed in extracts from patient P19, which correlated with the relatively high HCV RNA levels identified in the same liver (4 copies/hepatocyte, Figure 1B). Considering that hepatocytes represent 70–80% of the total liver cell mass [21], the estimated 40% cleavage of IPS1 observed in this patient could indicate that a relatively large fraction of hepatocytes were infected, unless IPS1 cleavage was caused by a cellular enzyme in uninfected cells in response to inflammation (see Discussion). It should be noted that the levels of HCV core were below the detection limit of our western blot analysis (even in patient P19) and hence, at least 10-fold lower than observed with HCV-infected tissue culture cells (Figure 1C). Thus, the results from protein analyses were consistent with the qRT-PCR results and suggested that in the majority of cases examined only a fraction of hepatocytes *in vivo* expressed HCV proteins.

### Activation of an IFN response in HCV-infected patients

**Figure 2:**
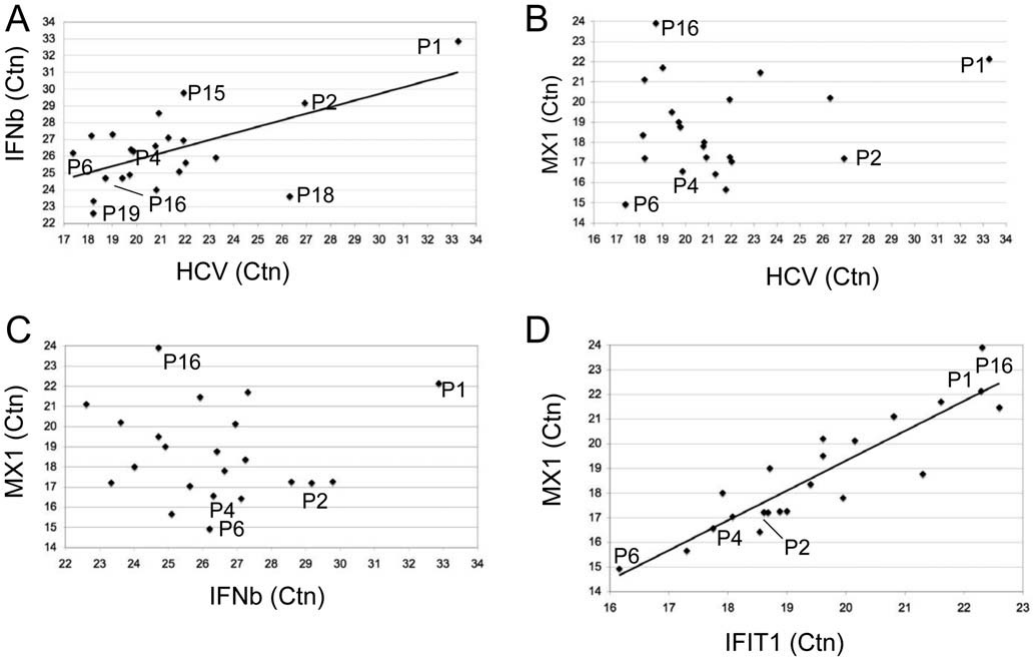
Correlation between HCV RNA and activation of IFNβ and ISGs. The Ct values for HCV, IFNβ, Mx1 and IFIT1 obtained from real-time qRT-PCR were plotted in different combinations as indicated. Ctn represents Ct values that were normalized with the Ct for albumin to account for the difference in cell number on each frozen section used for RNA isolation. Note, that Ct values and concentration of RNA are inversely related and that a difference of 1 in the Ct value corresponds to a two-fold difference in concentration.

DNA microarray studies with liver tissue from acutely HCV-infected chimpanzees and chronically infected human patients revealed that HCV replication induces interferon stimulated genes (ISGs) [11], [14]. Viral RNA and possibly, viral proteins induce IFNβ, which, in turn activates the IFN signal transduction pathway leading to the expression of ISGs [22], [23]. Whether the source of interferon responsible for ISG induction primarily is autocrine IFNβ from infected hepatocytes, paracrine IFNβ from vascular-associated non-parenchymal cells (e.g. liver sinusoidal endothelial cells, stellate cells, Kupffer cells, and portal myofibroblasts), or paracrine IFNγ produced by infiltrating lymphocytes remains unknown. To investigate whether a relationship existed between HCV RNA levels, IFNβ production and ISG expression, we determined RNA expression levels of IFNβ, IFIT1 and Mx1. All (21) HCV-infected patients examined in this study exhibited elevated IFNβ mRNA levels compared to the uninfected patient P1 (Figure 2A). The range of induction varied from about 8 in patient P15 to over 1000-fold in patient P19 compared with the uninfected patient P1. Viral load in the liver and expression of IFNβ expression correlated (p = 3.04E-3), suggesting that virus replication proportionally activated expression of the cytokine (see Discussion). Notably, the correlation between HCV RNA and IFNβ would be even stronger were the outlier P18 omitted from the regression analysis (p = 1.22E-4). In contrast, HCV RNA levels did not correlate with those of the ISG, Mx1 (Figure 2B). With one exception (P16), Mx1 levels were elevated in all patients compared to P1, although in 3 patients the increase was less than 2-fold (Figure 2B). Similar results were obtained when IFIT1 levels were used in lieu of Mx1, because the expression levels of the two ISGs correlated very well in individual patients (p<10E-4, Figure 2D). A similar correlation was observed between Mx1 and IFI27, a third ISG analyzed in this study (Figure S1). By contrast, levels of IFNβ and Mx1 induction did not correlate, suggesting that activation of ISGs is not directly controlled by IFNβ in HCV-infected livers (Figure 2C, see Discussion).

### Distribution of HCV and ISG RNA in liver

**Figure 3:**
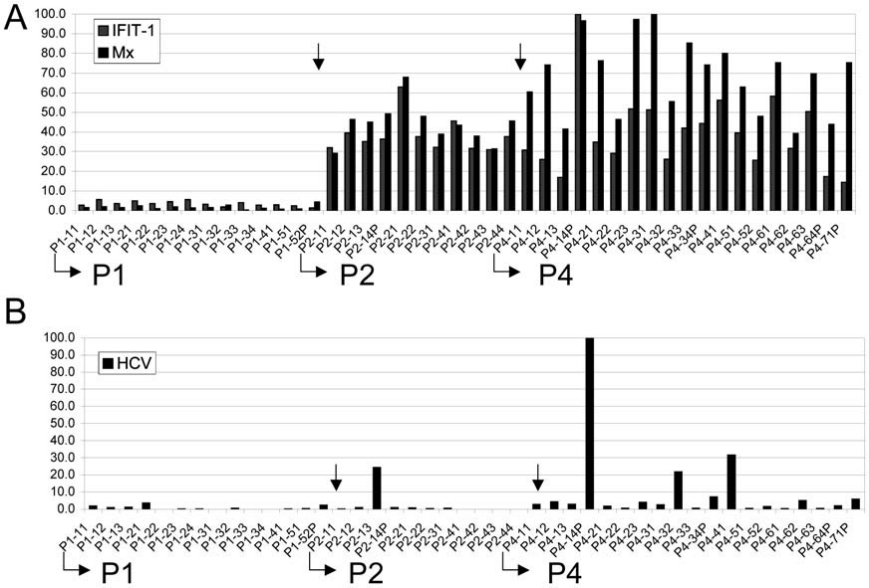
Focal accumulation of HCV RNA. A) The bar graph shows the levels of Mx1 and IFIT1 expression in groups of about 100 cells that were isolated by LCM from frozen section of patients P1, P2 and P4. Ct values obtained for the indicated genes were normalized with Ct for18S RNA. Expression levels are expressed on a scale from 0 to 100. B) Same as A except that the values for HCV RNA were displayed. The samples were sorted in order of the patients (P1, P2, P4) as indicated in the figure.

To determine the distribution of HCV and ISG RNA in liver sections, we used laser capture microdissection (LCM) to isolate small groups of about 100–200 hepatocytes and isolated RNA for real-time qRT-PCR analysis. To perform these investigations we selected frozen sections from patients P1 (control, uninfected), P2 and P4. Tissue sections from patients P2 and P4 did not exhibit fibrosis and were relatively large and, hence suitable for isolation of cells by LCM. Both patients underwent OLT approximately 4 months prior to the biopsy (Table 1). Because HCV RNA titers approach or surpass pre-transplantation RNA levels within a few days following surgery, intrahepatic RNA levels were at steady-state long before the biopsies were obtained [24], [25]. The estimated load of HCV in biopsies of infected liver grafts was 0.01 (P2) and 1.3 (P4) RNA copies per hepatocyte, respectively (Figure 1B). To investigate the distribution of HCV-infected cells and cells expressing Mx1, we performed real-time qRT-PCR analysis on the same RNA samples. The results showed that Mx1 expression levels were elevated to a similar degree in all samples obtained from HCV-infected patients P2 and P4 compared with the samples from HCV naive patient P1 (Figure 3A). Consistent with the results obtained from the analysis of whole tissue sections (Figure 2D), Mx1 RNA levels were on average higher in cells collected from patient P4 than from patient P2. In contrast to the observations made with Mx1, HCV RNA levels differed significantly among the samples analyzed (Figure 3B). For patient P2, only 1 of 11 (9%) samples analyzed exhibited significantly elevated HCV RNA levels, and for P4, 8 of 19 (42%) samples were positive (see Materials and Methods). Hence, the results clearly demonstrated that the presence of HCV RNA in infected liver tissue is focal, not uniform, as observed with ISGs Mx1 or IFIT1 (see Discussion).

## Discussion

The purpose of this study was to investigate the distribution of infected hepatocytes in chronic HCV infections and to determine whether a correlation exists between viral load in the liver and induction of IFNβ and ISGs. The results revealed that HCV infected hepatocytes are focally distributed in infected livers, explaining the surprisingly low average levels of HCV RNA in biopsy samples. Moreover, they provided evidence for a correlation between viral load and IFNβ expression, suggesting that viral replication could directly induce an antiviral state. However, the correlation between levels of IFNβ and ISG expression were insignificant, suggesting that control of the innate immune response during HCV infections depends on multiple factors.

Our study showed low average levels of HCV in the liver. Over 50% of tissue samples analyzed exhibited less that one copy of viral RNA per hepatocyte (Figure 1B). Patient P6 with the highest viral load among patients examined in this study (7–8 RNA copies/hepatocyte), still exhibited about 100-fold lower RNA levels than tissue culture cells expressing HCV replicons or infectious virus [5]. Consistent with these results, we observed, with one exception (P19, discussed below) only minor or no cleavage of IPS1, a known cellular target of the HCV protease (Figs. 1C and S1). Also, HCV core or NS5A proteins could not be detected in any of the patient samples analyzed so far, again reflecting the low average level of HCV replication in infected livers compared with replication in tissue culture cells (Figure 1C, results not shown). In tissue culture and livers of infected chimpanzees, the ratio between HCV plus to minus strands equals about 10 [4], [26]. Assuming a similar ratio in our patient cohort, near complete infection of hepatocytes could only have occurred with patient P6, provided that a single replication complex with one copy of minus strand DNA is sufficient to sustain an infection. In the majority of our patient cohort, 10% or fewer hepatocytes were infected. In this regard, our results were consistent with previous reports relying on qRT-PCR analysis (i.e. refs. [15], [16], [27]).

The hypothesis that focal replication could explain the low average levels of HCV RNA in infected livers was supported by our analysis of captured hepatocyte pools from frozen sections. Consistent with the very low RNA copy calculated for patient P2 (Figure 1B), less than 10% of the samples analyzed exhibited HCV levels that were significantly above background levels (Figure 3B). In the case of patient P4, who had higher average RNA levels compared with P2, 16% of the samples were strongly positive for HCV and another 26% were significantly elevated above background levels. In contrast to HCV, ISG levels were uniformly elevated in all samples from both patients. Therefore, we concluded that HCV is distributed in a piecemeal fashion in infected livers with infected and uninfected cells present at the same time (Figure 3B). We compared results from our qRT-PCR analysis with immunohistochemical analyses using paraformaldehyde fixed sections from patients P4, P6, and P9 incubated with NS5A-specific antibodies (Figure S2). The results were consistent with a model predicting focal distribution of HCV. However, similar experiments based on immunofluorescence were inconclusive due to high background caused by autofluorescence of liver tissue (results not shown).

How can we integrate these observations into a plausible model for natural HCV infections? An important issue concerns susceptibility of the hepatocyte population to HCV infection. Can all hepatocytes be infected or are some resistant? The latter possibility could be a consequence of selection of resistant hepatocytes during the course of chronic infections, which is characterized by continuous cell killing and regeneration of hepatocytes. We do not favor this possibility, because our analyses with small cell populations were performed with tissue samples from two patients (P2, P4) who underwent liver transplantation about 4 months before the biopsies were taken. Such a short time period is not sufficient for replacement of a large fraction of hepatocytes with a virus resistant population. A more plausible explanation would be that activation of an antiviral state either protects uninfected hepatocytes from *de novo* infection or induces clearance of infected hepatocytes. The latter possibility predicts that HCV replication in hepatocytes is transient *in vivo*. However, our results cannot definitively distinguish between the two models. They demonstrated that ISG expression is uniform in the liver (Figures 2 and 3A). Hence, they were consistent with previous reports demonstrating that ISG induction accompanies HCV infections independently of genotype, inflammation or fibrosis levels [11], [12], [13], [15], [27].

A major unresolved question concerns the mechanism responsible for ISG induction. Does infection of hepatocytes induce IFNβ or do resident endothelial cells, Kupffer cells, or infiltrating lymphocytes release IFNα/β or IFNγ [28] in response to HCV replication? While our experiments provided evidence for a positive correlation between intrahepatic HCV RNA and IFNβ expression levels (Figure 2A), and hence, indicated that infected hepatocytes are the primary inducer of IFNβ, they did not identify the cells producing the cytokine. The observation that the viral protease could cleave and inactivate IPS1, an upstream mediator of IFNβ production would indicate that non-parenchymal cells are the primary source of IFN production. On the other hand, it is equally possible that infected hepatocytes produce IFNβ because they might sense HCV immediately after infection, prior to accumulation of sufficient amounts of protease necessary to cleave and inactivate IPS1. Most likely, both, hepatocytes and infiltrating immune cells contribute to the expression of IFNβ observed in HCV infected patients. However, identification of the principal source of IFNβ production remains an important unresolved question.

The lack of a correlation between IFNβ and ISG production was unexpected. It is conceivable that the expression levels of IFNβ were very low and that the primary source of IFN in infected livers was IFN expressed from resident Kupffer cells or infiltrating lymphocytes. Also, such a mechanism would not require a strict correlation between viral replication and ISG induction, because recruitment of lymphocytes depends on many factors that can vary among patients including haplotype, diversity of the T and B cell repertoire and the production of chemokines and cytokines.

As noted above, we observed significant cleavage of IPS1 with protein extract from patient P19 (Figure 1C). Considering that normal liver consists of about 80% hepatocytes [21] and assuming that infiltrating lymphocytes account for at least 10% of total liver mass, the observed 50% cleavage of IPS1 would indicate that more than 70% of the hepatocytes were productively infected in this patient. However, this interpretation is inconsistent with the very low levels of core protein in the same sample, which could not be detected by western blot analysis (Figure 1C). Also, the results of another study demonstrating complete cleavage of IPS1 in some patient samples, are not consistent with a model explaining that IPS1 cleavage is limited to infected hepatocytes [29]. Instead, those results could be explained if IPS1 cleavage were mediated, at least in part, by a cellular protease that is activated by the inflammatory response independently of HCV infection. Hence, it is possible that cellular enzymes induced during inflammation account at least for some of the cleavage of IPS1 observed during HCV infections. Notably, it has just been reported that IPS1 is a substrate for certain caspases in cells induced to enter an apoptotic program [30].

In summary, this study, together with previous reports, demonstrated that HCV replication occurs in the face of an innate immune response that inhibits accumulation of viral RNA. Activation of ISGs could be important for maintaining chronic infections by preventing killing of infected hepatocytes either directly by the virus, or indirectly by cytotoxic T cells. The observation that HCV replicates in subpopulations of hepatocytes is most likely a consequence of ISG induction and invokes the possibility that HCV replication is transient in hepatocytes. Thus, chronic infections might depend on continuous cycles of infection and clearance of hepatocytes. Such a model would predict that inhibitors of *de novo* HCV infections would be effective for antiviral therapy. Thus, mechanisms controlling virus attachment, uptake, uncoating, and delivery of the viral genome to ribosomes might be excellent targets for novel antiviral drugs. Once identified, they could be combined with available protease and polymerase inhibitors and yield highly active antiviral therapies that might cure chronic HCV infections.

## Supporting Information

Table S1 (0.04 MB DOC)

Figure S1 Correlation between IFI27 and Mx1 expression. For an explanation of the figure see the legend to Figure 2.
(2.28 MB TIF)

Figure S2 NS5A expression in liver sections. Frozen sections were fixed with paraformaldehyde and incubated with NS5A monoclonal antibodies. Biotinylated antibodies against mouse IgG (DAKO, Inc.) were used as secondary antibodies. Tissue sections were incubated with peroxidase-labeled streptavidin and developed with 0.5 mg/ml of diaminobenzidine (DAB) in 0.03% hydrogen peroxide PBS. Sections were counterstained with hematoxylin, dehydrated in ethanol, and mounted with Permount.
(2.28 MB TIF)
