# Supplementary material for: Focal Distribution of Hepatitis C Virus RNA in Infected Livers
Source: PLoS One. 2009 Aug 18;4(8):e6661. doi: 10.1371/journal.pone.0006661 (PMC2722721; doi:10.1371/journal.pone.0006661)
Supplement: Table S1 — (0.04 MB DOC) [file pone.0006661.s001.doc]

**Table S1.** Assay identification (ID) for TaqMan primers from ABI.

| *Gene Name* | *ABI gene symbol* | *Gene Alias* | *Assay ID* |
| --- | --- | --- | --- |
| Eukaryotic 18s rRNA | 18S | n/a | Hs99999901_s1 |
| Albumin | ALB | n/a | Hs00609411_m1 |
| Interferon beta 1, fibroblast | IFNB1 | n/a | Hs00277188_s1 |
| Interferon alpha-inducible protein 27 | IFI27 | n/a | Hs00271467_m1 |
| interferon induced protein with tetratricopeptide repeats 1 | IFIT1 | IFI56, P56 | Hs00356631_g1 |
| myxovirus (influenza virus) resistance 1 | MX1 | MxA | Hs00182073_m1 |
| platelet/endothelial cell adhesion molecule (CD31 antigen) | PECAM1 | CD31 | Hs00169777_m1 |
| HCV | n/a | n/a | HCV_101005 |
| probe seq = 5’-accacaaggcctttcg-3’ |  |  |  |
| Forward primer = 5’-gactgctagccgagtagtgttg-3’ |  |  |  |
| rev primer = 5’-actcgcaagcaccctatcag-3’ |  |  |  |
|  |  |  |  |
|  |  |  |  |
