# Supplementary figures and images for: Focal Distribution of Hepatitis C Virus RNA in Infected Livers
Source: PLoS One. 2009 Aug 18;4(8):e6661. doi: 10.1371/journal.pone.0006661 (PMC2722721; doi:10.1371/journal.pone.0006661)

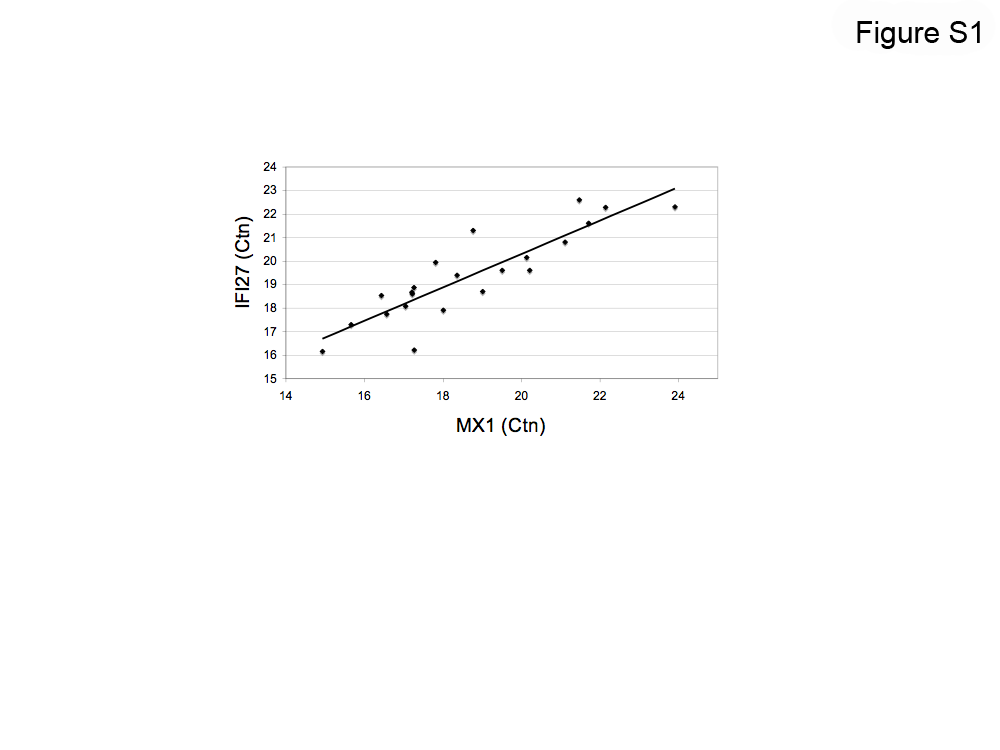

Supplement: Figure S1 — Correlation between IFI27 and Mx1 expression. For an explanation of the figure see the legend to Figure 2. (2.28 MB TIF) [file pone.0006661.s002.tif]

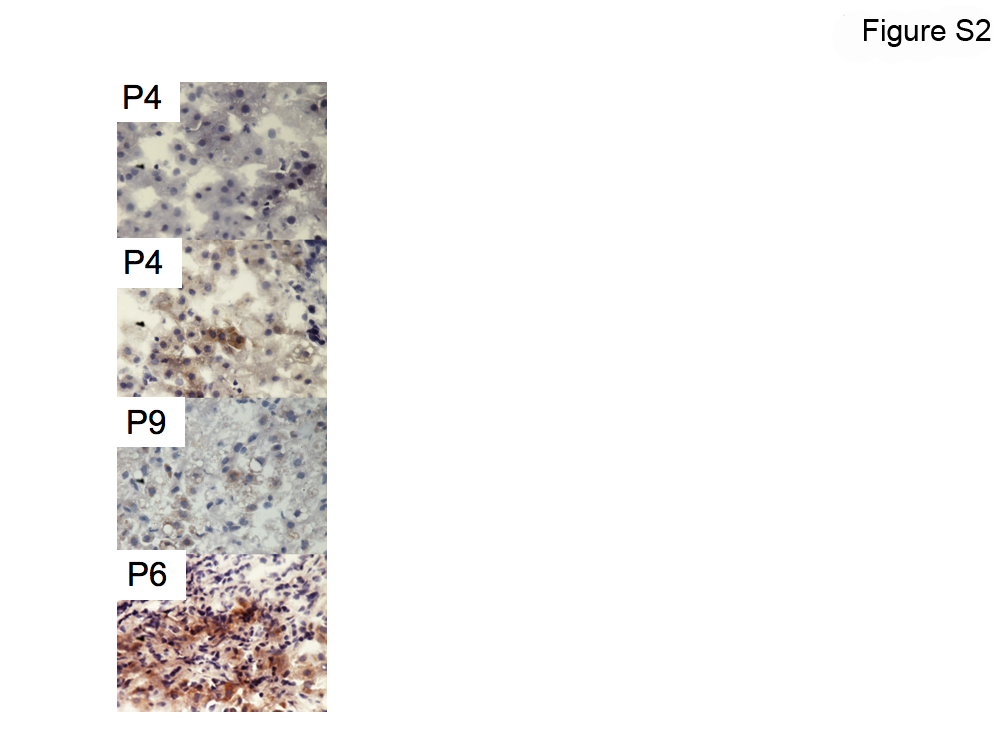

Supplement: Figure S2 — NS5A expression in liver sections. Frozen sections were fixed with paraformaldehyde and incubated with NS5A monoclonal antibodies. Biotinylated antibodies against mouse IgG (DAKO, Inc.) were used as secondary antibodies. Tissue sections were incubated with peroxidase-labeled streptavidin and developed with 0.5 mg/ml of diaminobenzidine (DAB) in 0.03% hydrogen peroxide PBS. Sections were counterstained with hematoxylin, dehydrated in ethanol, and mounted with Permount. (2.28 MB TIF) [file pone.0006661.s003.tif]
